# Testing the Stress-Gradient Hypothesis at the Roof of the World: Effects of the Cushion Plant *Thylacospermum caespitosum* on Species Assemblages

**DOI:** 10.1371/journal.pone.0053514

**Published:** 2013-01-10

**Authors:** Miroslav Dvorský, Jiří Doležal, Martin Kopecký, Zuzana Chlumská, Kateřina Janatková, Jan Altman, Francesco de Bello, Klára Řeháková

**Affiliations:** 1 Section of Plant Ecology, Institute of Botany, Academy of Sciences of the Czech Republic, Třeboň, Czech Republic; 2 Department of Botany, Faculty of Science, University of South Bohemia, České Budějovice, Czech Republic; 3 Department of Vegetation Ecology, Institute of Botany, Academy of Sciences of the Czech Republic, Brno, Czech Republic; WSL Institute for Snow and Avalanche Research SLF, Switzerland

## Abstract

Many cushion plants ameliorate the harsh environment they inhabit in alpine ecosystems and act as nurse plants, with significantly more species growing within their canopy than outside. These facilitative interactions seem to increase with the abiotic stress, thus supporting the stress-gradient hypothesis. We tested this prediction by exploring the association pattern of vascular plants with the dominant cushion plant *Thylacospermum caespitosum* (Caryophyllaceae) in the arid Trans-Himalaya, where vascular plants occur at one of the highest worldwide elevational limits. We compared plant composition between 1112 pair-plots placed both inside cushions and in surrounding open areas, in communities from cold steppes to subnival zones along two elevational gradients (East Karakoram: 4850–5250 m and Little Tibet: 5350–5850 m). We used PERMANOVA to assess differences in species composition, Friedman-based permutation tests to determine individual species habitat preferences, species-area curves to assess whether interactions are size-dependent and competitive intensity and importance indices to evaluate plant-plant interactions. No indications for net facilitation were found along the elevation gradients. The open areas were not only richer in species, but not a single species preferred to grow exclusively inside cushions, while 39–60% of 56 species detected had a significant preference for the habitat outside cushions. Across the entire elevation range of *T. caespitosum,* the number and abundance of species were greater outside cushions, suggesting that competitive rather than facilitative interactions prevail. This was supported by lower soil nutrient contents inside cushions, indicating a resource preemption, and little thermal amelioration at the extreme end of the elevational gradient. We attribute the negative associations to competition for limited resources, a strong environmental filter in arid high-mountain environment selecting the stress-tolerant species that do not rely on help from other plants during their life cycle and to the fact the cushions do not provide a better microhabitat to grow in.

## Introduction

The response of alpine and subnival plants to recent climate warming and their potential distributional shifts along elevational gradients may, to a large extent, hinge on interactions between species [1]. With rising temperature, many species from the lower alpine zone have been recently shown to extend their range limits toward the subnival zone [2]–[4]. This process can be facilitated or hindered by resident species. Newly arrived species to upper alpine zones do not need to be well adapted to harsh conditions and their survival may depend on the presence of safe microsites [5]. Among protected (nurse) habitats which could potentially provide better growing conditions within the alpine zone, are canopies of dominant cushion plants. These low-growing and compact species represent one of the most suitable survival strategies in the alpine and arctic zones. Here, plants must cope with multiple stress factors including low temperature, strong wind and abrasion, unstable substrate and solifluction, and low nutrient and water availability [6]. Under such conditions, the main mechanisms behind the facilitative processes could be thermal amelioration [7], [8], water and nutrient provision [8]–[10] and protection from strong desiccant winds by cushion plants [8], [11].

Many cushion plant species have been shown to ameliorate the harsh environment and enable other plants to establish, survive and perform better in environments where they would otherwise not succeed [8], [12]–[17]. For example, a literature search of studies assessing potential nursing effects of cushions on other plants resulted in 27 key publications (Table 1), all showing predominantly facilitative effects. The research on positive plant interactions with cushion species significantly helped to verify the so-called stress-gradient-hypothesis (SGH). This model predicts that the outcome of plant interactions depends on the severity of the physical environment, with positive effects (facilitation) being more important in stressful environments and resource competition prevailing in less harsh conditions [18]. The positive role of cushion plants in enhancing the local community diversity has been mainly documented in temperate alpine and arctic conditions. Conversely, recent studies conducted in arid and semi-arid mountains [19]–[22] reported non-positive or negative interactions under extreme conditions of low temperature and water deficit [8], [16], [23]. This supported the theoretical prediction that high stress could minimise the importance of facilitative effects by exclusively selecting the stress-tolerant species that do not rely on help from other plants during their life cycle. A debate between ecologists [19]–[21] resulted in a refined SGH [24] in which the predicted results of plant-plant interactions are specified with respect to the type of abiotic stress (resource vs. non-resource limitation) and the relative strategy of the participating species (competitive vs. stress tolerant) [25].

**Table 1:** List of studies exploring effects of cushion species on other plants.

Study	Year	Species	Altitude (m a.s.l.)	region	temperature regime	rainfall	effect
**Pyšek a Liška**	1991	*Sibbaldia tetrandra*	3800	Pamiro-Alai, 42°N	-	714	+
**Nuňez et al.**	1999	*Mulinum leptacanthum, Oreopolus glacialis*	1550–1600	Andes, 41°S	A 3°C	1000	+
**Badano et al.**	2002	*Oreopolus glacialis*	1900	Andes, 37°S		2300	+ but species specific
**Cavieres et al.**	2002	*Bolax gummifera*	700–900	Andes, 50°S	A 5°C	900–1000	+ and more evident in higher altitudes
**Arroyo et al.**	2003	*Azorella monantha*	700–900	Andes, 50°S	A 5°C	900–1000	+ and great altitudinal variation
**Cavieres et al.**	2005	*Azorella monantha*	3100–3300	Andes, 33°S	3.0°C April, 7.6°C February	943	+
**Acuňa-Rodriguez et al.**	2006	*Laretia acaulis*	2800	Andes, 33°S	A 5.4°C, S 10°C	400–900	+
**Badano et al.**	2006	*Azorella monantha*	3580–3630	Andes, 33°S	S 4°C, 4–5 months	>900	cushion added new species into the community
**Badano & Cavieres**	2006a	*Azorella monantha, Azorella madreporica, Adesmia subterranea*	3200–4000	Andes, 30–33°S	A 4.6°C (3700 m)	242–900	+
**Badano & Cavieres**	2006b	*Pycnophyllum bryoides, Azorella madreporica, Adesmia subterranea, Azorella monantha, Laretia acaulis, Mulinum leptacanthum, Oreopolus glacialis, Discaria nana*	1600–4400	Andes, 23–41°S	–		+
**Cavieres et al.**	2006	*Laretia acaulis*	2800–3200	Andes, 33°S	S 7–12°C (2600 m), 3–7.6°C (3150 m)	445–943	increased facilitation at lower (drier) site
**Zoller & Lenzin**	2006	*Eritrichium nanum*	2170–3320	Alps	–	2170–3320	+
**Cavieres et al.**	2007	*Laretia acaulis, Azorella monantha*	2800–3600	Andes, 33°S	S 10°C (2800 m), ca. 4°C (3600 m)	400–900	+
**Badano & Marquet**	2008	*Azorella monantha*	3580–3630	Andes, 33°S	S 4°C (3600 m)	>900	+
**Fajardo et al.**	2008	*Azorella madreporica*	3580–3630	Andes, 33°S	S 3–7.6 °C	943	+/−
**Antonsson et al.**	2009	*Silene acaulis*	1150–1447	Scandes, 68°N	A –2.0 °C, warmest month mean 8.6 °C	839	+ above a certain altitudinal threshold
**Arredondo-Núňez et al.**	2009	a meta-analysis	700–4400	–	–		+
**Badano & Marquet**	2009	*Azorella madreporica*	3400	Andes, 33°S	S 6°C, 4–5 months	>900	+
**Cavieres & Badano**	2009	*Mulinum leptacanthum, Discaria nana, Bolax gummifera, Azorella monantha, Pycnophyllum bryoides, Azorella madreporica, Adesmia subterranea, Laretia acaulis, Oreopolus glacialis*	900–4400	Andes, 23–50°S	S 2.6–10.1°C	42–1117	always+but lower effects at both extremes of the environmental severity gradient.
**Haussmann et al.**	2009	*Azorella selago*	300	Marion Island, 46°S	A 6°C	2000	
**Quiroz et al.**	2009	*Azorella madreporica*	3200–3580	Andes, 33°S	W 1.7°C, S 6.8°C (3,150 m)	400	+ and changes with altitude
**Sklenář**	2009	*Azorella dicranoides, Azorella corymbosa, Eudema nubigena, Hypochaeris sp., Xenophyllum humile*	4650	Andes, 0°S	–	no data	+
**Badano et al.**	2010	*Azorella madreporica, Laretia acaulis,*	2700–3600	Andes, 33°S	S 7–12°C (2600 m)	400–900	+/− depends on the environmental context
**Haussmann et al.**	2010	*Azorella selago*	300	Marion Island, 46°S	A 6°C	2000	+
**Yang et al.**	2010	*Arenaria polytrichoides*	4500–4700	Himalaya, 28°N	A –1.0°C, S 5.6°C	680–790	+ and increases with altitude
**de Bello et al.**	2011	*Thylacospermum caespitosum*	5900	Transhimalaya, 33°N	A –10.7°C, S 4.2°C,	100	-
**Anthelme et al.**	2012	*Azorella aretioides*	4400, 4550, 4700	Andes, 0°S	1.15°C,	1000	- lower elevation,+upper elevation
**this study**		*Thylacospermum caespitosum*	4850–5850	Transhimalaya, 33°N	A –10.4 to –1.6°C, S 4.4 to 7.7°C	100	–

For each study, the table indicates the cushion species that was studied, the elevational range considered (in m a.s.l.), the study region, the approximate mean yearly rainfall (mm year^−1^), mean annual (A), summer (S), winter (W) temperatures, and the effect detected (‘+’ indicates facilitation, ‘–’ indicates negative effects). Based on de Bello et al. [22].

The positive role of cushion plants has been mostly documented in mountains with elevations below 5000 m and with an abundant water supply [9], [15], [16], [26], [27]. The type of plant interactions that can be expected under higher elevations (5000–6000 m) and aridity (<100 mm per year) is, however, largely unknown. Such conditions exist in the dry mountains of Trans-Himalaya, where vascular plants grow continuously up to 6000 m. Recently, we have documented that cushions of *Thylacospermum caespitosum* (Caryophyllaceae) do not facilitate other plants at extremely high elevations (5900 m) in the dry mountains of eastern Ladakh [22]. These findings led us to hypothesise that positive interactions between species could be more prominent under moderately stressful than extreme conditions [28]. This called for further testing of the cushion’s nursing effect along its entire distribution range.

Here we tested the nursing effect of cushion species *Thylacospermum caespitosum* (Cambessèdes) Schischkin (Caryophyllaceae) by assessing plant associations along an unprecedented elevational gradient (4850–5850 m) in the arid mountains of NW Himalayas. Despite the importance of cushion plants in alpine ecosystem [8]–[17], their impacts and functions require further attention, especially in remote mountain regions such as the Himalayas. This high altitude region is being strongly impacted by climate change and is experiencing rapid changes in biodiversity [29]. These changes in biodiversity can alter ecosystem processes and the resilience and resistance of ecosystems to environmental change [6]. Without baseline data on plant-plant interactions, however, we cannot track the effects of climate change, and without an understanding of the drivers of community assembly, we cannot predict how climate change may affect these high-altitude plant communities.

We investigated which of the two contrasting predictions would apply: would the nurse effect take place, as in most of the key studies on cushions worldwide, or would we find no facilitation as implied by the refined stress-gradient hypothesis? Specifically, we asked the following questions: 1) Are other vascular plants positively or negatively associated with cushions of *T. caespitosum*? 2) Does the effect of *T. caespitosum* on the surrounding vegetation depend on its size? 3) Does *T. caespitosum* influence the surrounding abiotic environment (temperature and soil physico-chemical properties)? 4) Do these effects and patterns change with elevation and water availability?

## Methods

### Study Region

**Figure 1:**
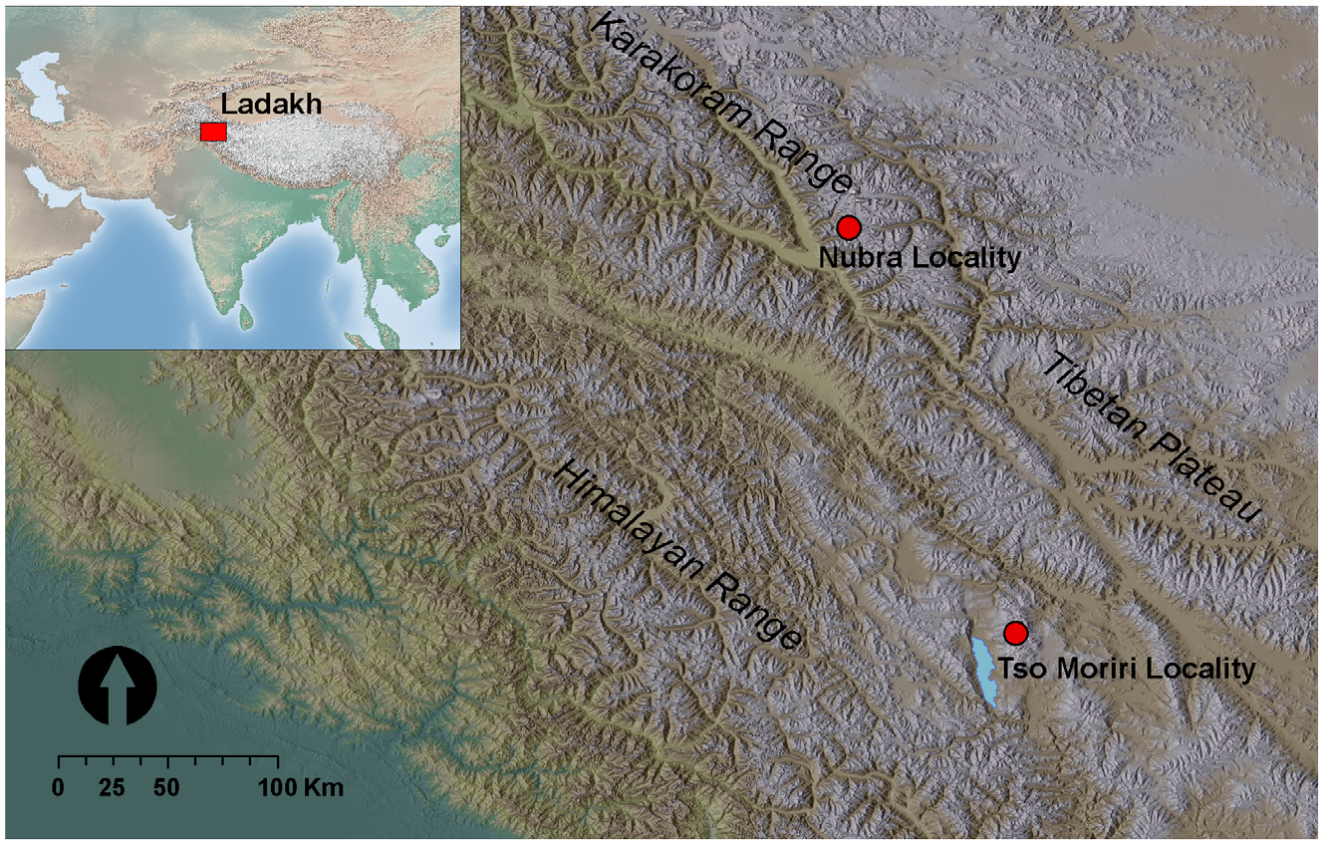
Location of the two study localities. Nubra is situated within E Karakoram Range, Tso Moriri belongs to Tibetan Plateau.

The study region is situated in Ladakh, Jammu & Kashmir State, India (Figure 1). The area is a part of the Trans-Himalaya, being delimited by the Eastern Karakoram Range in the north and by the Great Himalaya Range in the south. Due to its position in the rain-shadow of the Himalaya Range, the region is arid and receives very little precipitation (<100 mm) [30], [31]. Evaporation exceeds precipitation at lower and middle elevations. At elevations above 5300 m, precipitation tends to increase [32], however above 5600 m, water may remain unavailable due to soil water freezing.

**Figure 2:**
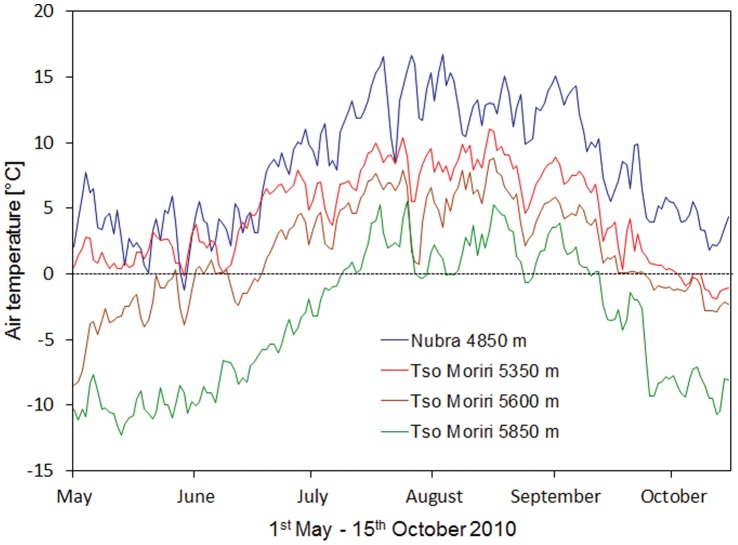
Changes in air temperature regime with increasing elevation. Shown are mean daily air temperatures during the summer season of 2010 for selected Nubra and Tso Moriri sites.

To test the generality of plant interactions, we analysed the associations between *T. caespitosum* and other vascular plants from the cold steppes up to the subnival zones in two mountain ranges (Eastern Karakoram, Little Tibet). These differ in overall glaciation and hence have different upper distributional limits of vascular plant existence [33], [34]. The two locations enabled us to test the SGH along an elevational gradient, mainly associated with decreasing temperature (Figure 2), which together covered the entire elevation range of *T. caespitosum* cushions in Ladakh. This is correlated with a significant decrease in the mean annual/summer temperatures (from –1.6/7.7°C to −10.4/4.4°C between 4850–5850 m).

The first locality was a side valley near the village of Tiggur in Nubra Valley, which is situated in the northern part of Ladakh (34°45′N, 77°35′E) and belongs geomorphologically to the Eastern Karakoram Range (with the highest peak of Saser Kangri 7672 m, 15 km north of our study site). The relief is characterised by sharp and rugged ridges. Valleys are narrow and steep and regularly end in glaciers, the fronts of which usually start at c. 5300 m. The bedrock consists mostly of Nubra-Siachen batholith and leucogranites [35]. In a wetter and stronly glaciated Karakoram, the cushions descend to lower elevations compare to dry Little Tibet where the cushions ascend to 5900 m due to a poor glaciation and relatively well developed soils at high elevations [33]. Zonation of the vegetation is similar to the Little Tibet, but the vegetation zones are shifted downwards by 300–400 metres because of higher elevation of surrounding mountains and their large-scale glaciation leading to development of rather extensive alpine grasslands around glacial lakes and morianes at 5000–5200 m.

The second locality is the valley of the Lupgo stream on the western side of Chamser Kangri Peak, near Tso Moriri Lake; this belongs to Changtang region in the eastern part of Ladakh (32°59′N, 78°24′E). This area of the Trans-Himalaya can be characterised as a high-elevation plain, being the westernmost spur of the Tibetan Plateau (also called Little Tibet). It is of relatively flat relief with gentle slopes bearing many rounded and poorly glaciated peaks, reaching 6666 m (Lungser Kangri). The substrate ranges from siliceous rocks (Precambrian granites, Tso Moriri gneiss) to calcareous and saline sediments [35]. At lower elevations much of the area is covered by cold desert and semi-desert vegetation, a steppe zone is found at middle elevations up to c. 5000–5400 m, alpine grasslands form a narrow belt above the steppe zone and subnival vegetation is developed at the highest elevations of 5700–6000 m [33], [34]. The snow line, in its climatological sense [36], is situated at 6100–6200 m, i.e. coinciding with the greatest recorded elevation of vascular plants in the region (c. 6150 m).

### Target Species

The target species of this study, *T. caespitosum*, is one of the most prominent high-alpine cushion plants in the Himalayas. It is a perennial plant with a woody taproot and it forms very dense and solid cushions [37]. Although very little is known about its speed of growth or longevity, the largest cushions in the study region can be more than 150 cm in diameter and may persist for decades or even centuries [38], [39]. In the study region, *T. caespitosum* occurs in the elevational range of 4600–5900 m [33], [37]. In Nubra, *T. caespitosum* can be found from 4600 to 5480 m, from dry alpine steppes at lower elevations (with poorly developed sandy soils dominated by *Tanacetum tibeticum*, *Artemisia minor* and *Elymus schrenkianus*) to rocky outcrops at higher elevations surrounded by glacier moraines and mesic alpine meadows (dominated by *Potentilla pamirica, Poa attenuata*, and *Astragalus confertus*). In the Tso Moriri locality, the cushions occur from 5100 to 5960 m, from dry alpine screes at lower elevations (dominated by *Poa attenuata*, *Urtica hyperborea* and *Dracocephalum heterophyllum*), to the subnival zone with poorly developed soils covered by algal crusts and few vascular plants (*Saussurea gnaphalodes*, *Draba altaica* and *Stellaria decumbens*). Plant scientific names follow Klimeš and Dickoré [40].

### Vegetation Sampling

At both localities, 4 sites along an elevational gradient were selected to cover the entire elevation range of *T. caespitosum*: in Nubra Valley at 4850 m, 5000 m, 5100 m and 5250 m and in Tso Moriri at 5350 m, 5600 m, 5750 m and 5850 m. At each elevational site, we systematically surveyed cushions within an area of ca. 1 ha so that we received sufficient replications (in Nubra n = 66, 61, 69, 77; in Tso Moriri n = 70, 70, 70, 73). The surveyed cushions represented all size classes with a diameter range of 4–132 cm. However, the most common size class was 40–60 cm in Nubra (n = 79) and 20–40 cm in Tso Moriri (n = 102). Two vegetation samples were taken at each cushion: one covering the cushion itself, the other one had the same size and shape and was marked with a flexible wire ring which was placed randomly in the open site outside the cushion at a distance equalling the cushion diameter. If the spot was occupied by another cushion or a stone covering the whole area of the sample, the sample was taken on the opposite side of the cushion. We recorded vascular plant species rooting within the respective sample areas and their percentage cover. At all sites, *T. caespitosum* was one of the few dominant species [33]. Its relative cover increased with increasing elevation (from 5 to 15%, based on data from 44 square plots 100 m^2^ in size, sampled between 4700–5850 m, [33], [37]). The total vegetation cover ranged from 10 to 40%. The fieldwork was carried out during the peak of the vegetation season (August) in 2009–2011. No specific permits were required for the described field studies, the locations were not privately-owned or protected in any way and the field studies did not involve endangered or protected species.

### Microclimatic Measurements

We recorded air temperature and relative air humidity using datalogger HOBO U23 Pro v2 placed 10 cm above the soil surface and shielded against direct sunlight. The measurements were recorded every two hours from August 2009 to August 2011. Additionally, at three sites (Nubra 5000 m, Tso Moriri 5600 m and 5850 m), we chose one cushion of average size and placed a temperature logger (iButton® DS1923, Maxim Integrated Products) in the soil below it and another one in the soil of the adjacent open area 50 cm from the cushion. Inside the cushions, the loggers were placed 2 cm deep in the substrate under the cushion tissue, where the colonising species were thought to be rooting. In the open areas, the loggers were burried 2 cm under the soil surface. The measurements were recorded every three hours from September 2009 to August 2010.

### Soil Sampling

During August 2009, we collected soil samples for supplementary physico-chemical analyses from 6 cushions within the most common size class at each elevational site (96 samples in total). We took one soil sample (150 g) below a cushion and one sample in the open site. The samples were air-dried for 10 h on an aluminium plate, placed in sterile 540 ml polypropylene bags (Nasco Whirl-Pak®). In the laboratory, the samples were weighted, oven-dried at 100°C, ground in a mortar and sieved to 2 mm fraction after the removal of roots. The analysed components included total nitrogen (TN), NH_4_^+^-N, NO_3_^–^N, PO_4_^−3^-P, Ca^2+^, Mg^2+^, Na^+^, K^+^, pH, texture (percentage content of particles larger than 0.5 mm), gravimetric water content (GWC) and organic matter (OM). Soil physico-chemical analyses were conducted in accordance with the standardised methods of the Association of German Agricultural Analytical and Research Institutes (VDLUFA 1991). Soil pH was potentiometrically measured in a suspension with 0.01 M CaCl_2_. Plant available N and P content of the soil samples was analysed colorimetrically with an FIAstar 5010 Analyzer (Foss Tecator AB, Höganäs, Sweden) and the cations with AAS (SpectrAA 640, Varian Techtron, Melbourne, Australia). Soil moisture was futher measured at each soil sampling point as percent volumetric water content (VWC) using HydroSense Measurement System (Campbell Scientific, Australia).

### Data Analyses

In all statistical analyses, the two localities (Nubra and Tso Moriri) were treated separately as they represent a separate gradient going from a cold steppe zone to a subnival zone. Further in the text, we distinguish two vascular plant habitats: 1) inside cushions and 2) open areas outside cushions.

To assess whether the species richness of vascular plants associated with cushions differed from those in open areas, we calculated sample-based rarefaction curves (a measure of total species richness or species pool) [41] separately for each habitat and elevation site. The curves were computed as means of 9999 sample-based species accumulation curves that resulted from the random ordering of all plots belonging to each habitat type. The calculation was performed with the Juice program [42].

To assess whether the number of species increases with the sample area (i.e. cushion size) and to compare these species-area relationships between cushions and open areas, we fitted a multiple linear model with log(species richness) as the dependent variable, log(sample size) as the continuous predictor and the sample position as the categorical predictor variable. This was done for each study site. We additionally performed a test for differences in slope and intercept parameter estimates between the two regression lines as implemented in the *smatr* package [43] for R software [44]. The significant deviation from the common intercept indicated differences in species richness per unit area between cushions and open areas. The contrasting slopes (*test of parallelism*) indicated a significant difference in the rate at which species richness increased with enlarging area.

To explore whether species composition of vascular plants growing on cushions differed from those in open areas, we performed a non-metric multidimensional scaling (NMDS) on a Bray-Curtis dissimilarity matrix, which was calculated from square-root transformed percentage cover data standardised by sample totals. We ran NMDS in two dimensions and used several random starts in order to achieve the optimum configuration. The results were visualised by an ordination diagram with 95% confidence ellipses around multivariate centroid of samples from each habitat type. We further used permutational multivariate analysis of variance (PERMANOVA) to test the differences in species composition between the cushion habitat and open areas [45]. We assessed the significance of the cushion effect by a nonparametric test with 999 permutations restricted within the blocks (i.e. a pair of neighboring cushion/non-cushion samples). NMDS and PERMANOVA were calculated separately for each elevation site using *metaMDS* and *adonis* functions from vegan library for R software [46].

To assess whether individual plant species were significantly more associated with cushions or with open areas, we used the Friedman test, based on 9999 Monte-Carlo resamplings [47]. This is a nonparametric test assessing the symmetry of responses across treatments for repeated measures or split-plot data. The two positions (i.e. within and outside cushions) were considered as repeat measures for each individual cushion. The Friedman test was applied to presence/absence as well as abundance (percent cover) data in each species. We analysed the degree of association of each species with cushions for each elevation site to evaluate whether there is an increasing number of facilitated species with elevation. Analyses were run using the *coin* package [48] for R software.

In order to quantify the outcome of interactions between *Thylacospermum* and other species, we calculated two widely used plant-plant interaction indices: (1) RII - relative interaction intensity index [49], and (2) I_imp_ - relative importance index [50]. These are reffered to as competitive intensity and importance and the index values range from competition (−1) to facilitation (+1) and are symmetrical around 0. They can also be scaled up to measure interactions at both individual and community levels within each elevation site. We calculated RII and I_imp_ values at the community level by pooling individual values of all species in vegetation samples. As for individual species both indices yielded similar results, we present only the RII values (see Figure S1).

In order to reveal differences in the soil physico-chemical parameters between cushions and open areas and their dependence on elevation, we used linear mixed-effect models or generalised linear mixed-effect models, depending on the nature of a particular response variable (assuming Gaussian, quasi-Poisson, or quasi-binomial distributions). The pair-samples represented a random effect factor, and elevation and position (cushions and open areas) were fixed effect factors. The tests were based on the likelihood-ratio approach, approximating the difference in model deviances with a χ^2^ distribution. To control for familywise error rate, the false discovery rate procedure was performed [51]. Analyses were run using the *lme4* package [52] in R software.

## Results

### Species Richness

**Figure 3:**
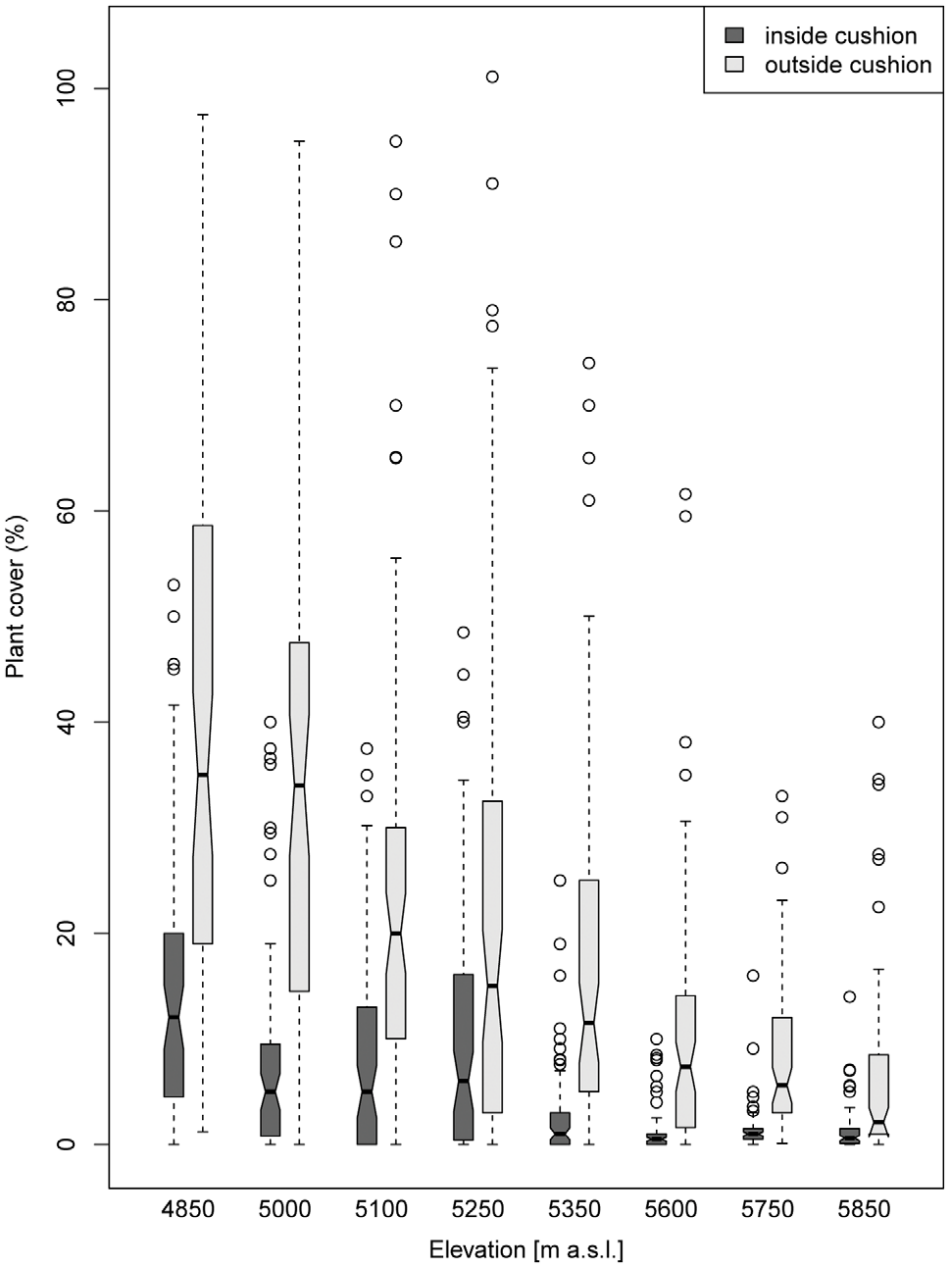
Comparision of total cover of vascular plants growing on the *Thylacospermum caespitosum* cushions and open areas outside cushions (vegetated area, the rest is bare soil or soil crusts) in Nubra (4850–5250 m) and Tso Moriri (5350–5850 m). The surveyed cushions represented all size classes with a diameter range of 4–132 cm. Boxes represent 25–75% of values, black dots are medians, whiskers are 1.5 interquartile ranges and open dots are outliers. Notches in the boxes indicate the significance of between-group differences: if notches of two groups do not overlap this is strong evidence that these groups differ significantly.

In total, 56 species were recorded (39 in Nubra, 30 in Tso Moriri, Tables 2 and 3), belonging to 15 families. The richest family at both localities was Asteraceae (nine species in Nubra, six in Tso Moriri). Fourteen species (25%) were common for both localities.The total percentage cover of plant species growing on bare ground outside cushions was significantly higher than that of species established inside cushions (Nubra: 30.4 vs 10.9%, Tso Moriri: 10.6 vs 1.8%). The total cover outside cushions decreased significantly with increasing elevation at both localities (Nubra: from 41 to 23% between 4850–5250 m, Tso Moriri: from 14 to 8% between 5350–5850 m), while non-significant elevation differences were found for the values inside cushions (Figure 3).

**Table 2:** Species frequency of occurrence inside (in) and outside (out) cushions of *T. caespitosum* at four elevation sites in Nubra.

Species	Family	Clona-lity	Life	4850 m				5000 m				5100 m				5250 m				
			form	in	out	F^p^	F^a^	in	out	F^p^	F^a^	in	out	F^p^	F^a^	in	out	F^p^	F^a^	total
Poa attenuata	Poa	yes	H	34	50	***	***	24	39	***	***	19	30		**	30	41		**	267
Potentilla pamirica	Rosa	no	H	0	8	**	*	14	28	**	**	18	26		***	31	29			154
Oxytropis tatarica	Faba	no	H	0	0			1	5			23	32		*	20	32	*	*	113
Astragalus strictus	Faba	no	H	30	45	**	***	11	23	*	**	0	1	***	***	0	0			110
Astragalus confertus	Faba	no	H	0	0			6	12		*	15	25		*	19	24		*	101
Draba altaica	Brassica	no	Ch	0	2			3	18	***	***	10	16			22	30			101
Leontopodium leontopodinum	Aster	yes	H	19	21			7	18	**	***	4	2			4	4			79
Oxytropis densa	Faba	no	H	34	34			5	6			0	0			0	0			79
Artemisia minor	Aster	no	Ch	12	37	***	***	11	16			0	0			0	0			76
Carex pseudofoetida	Cyper	yes	H	10	13		**	9	8			1	2			10	10			63
Tanacetum tibeticum	Aster	no	Ch	5	9			4	11		*	5	15	*	*	4	5			58
Potentilla bifurca	Rosa	yes	H	15	21			4	4			0	0			0	0			44
Lloydia serotina	Lili	yes	G	0	0			4	3			0	1			18	15			41
Elymus schrenkianus	Poa	yes	H	8	7			0	0			5	4			1	0			25
Potentilla gelida	Rosa	no	H	0	0			6	18	***	***	0	0			0	0			24
Potentilla multifida agg.	Rosa	no	H	4	10			1	2			0	0			0	0			17
Oxytropis platysema	Faba	no	H	0	0			7	7			0	0			1	1			16
Saussurea glacialis	Aster	no	H	0	0			1	0			4	1			5	5			16
Taraxacum sp.	Aster	no	H	0	0			3	10	*		0	0			1	1			15
Trisetum spicatum	Poa	yes	H	0	0			6	6			0	0			1	0			13
Oxytropis chiliophylla	Faba	no	H	0	0			3	8	*	*	0	0			0	0			11
Carex sp.	Cyper	yes	H	5	4			0	0			0	0			0	0			9
Gentianella azurea	Gentiana	no	T	1	4			0	0			0	0			0	4			9
Artemisia santolinifolia	Aster	no	Ch	0	8	***	***	0	0			0	0			0	0			8
Elymus schugnanicus	Poa	yes	H	3	4			0	0			0	0			0	0			7
Braya humilis	Brassica	no	H	0	0			1	4			0	0			0	1			6
Thalictrum alpinum	Ranuncul	yes	H	0	0			1	1			0	0			1	3			6
Aster flaccidus	Aster	yes	H	0	0			0	0			0	0			1	2			3
Ephedra gerardiana	Ephedra	yes	Ch	2	1			0	0			0	0			0	0			3
Oxytropis pusilla	Faba	no	H	0	2			0	0			0	0			0	1			3
Saussurea gnaphalodes	Aster	no	H	0	0			0	0			2	0			1	0			3
Urtica hyperborea	Urtica	no	H	0	2		***	1	0			0	0			0	0			3
Potentilla saundersiana	Rosa	no	H	0	2			0	0			0	0			0	0			2
Waldheimia tridactylites	Aster	no	H	0	0			1	1			0	0			0	0			2
Carex borii	Cyper	yes	H	0	0			0	0			0	0			0	1			1
Kobresia schoenoides	Cyper	yes	H	0	0			0	0			0	0			1	0			1
Lomatogonium thomsonii	Gentiana	no	T	0	1			0	0			0	0			0	0			1
Sibbaldia tetrandra	Rosa	yes	Ch	0	0			0	0			0	0			0	1			1
	total			182	285	5	7	134	248	8	9	106	155	2	6	171	210	1	3	
	average richness			2.8	4.3			2.2	4.1			1.5	2.3			2.2	2.8			
	total richness			14	21			24	22			11	12			18	19			

Friedman-based resampling on presence/absence (F^p^) and cover-abundance (F^a^) data was used to define the preferred position of a species (*** P<0.001, ** P<0.01, * P<0.05). All significant preferences refer to habitat outside cushion. Also shown is potential for clonal growth and life form (H – hemicryptophyte, Ch – chamaephyte, G – geophyte, T - terophyte).

**Table 3:** Species frequency of occurrence inside (in) and outside (out) cushions of *T. caespitosum* at four elevation sites in Tso Moriri (see Table 2 for explanation).

Species	Family	Clona-lity	Life	5350 m				5600 m				5750 m				5850 m				
			form	in	out	F^p^	F^a^	in	out	F^p^	F^a^	in	out	F^p^	F^a^	in	out	F^p^	F^a^	total
Poa attenuata	Poa	yes	H	15	26	**		34	46	*	**	43	49		***	27	43	**	**	283
Draba altaica	Brassica	no	Ch	0	0			20	39	**	**	32	64	***	***	29	50	***	***	234
Saussurea gnaphalodes	Aster	no	H	1	2			12	20	*	***	16	29	**	**	24	38	*	*	142
Desideria pumila	Brassica	no	H	0	0			3	7			22	21			10	13			76
Stellaria decumbens	Caryo	no	H	0	0			0	3			0	28	***	***	0	26	***	***	57
Artemisia minor	Aster	no	Ch	17	37	***	**	0	0			0	0			0	0			54
Waldheimia tridactylites	Aster	no	H	0	0			6	17	**	**	0	0			8	13		*	44
Eritrichium hemisphaericum	Boragin	no	H	0	0			0	10	***	***	0	9	**	**	1	15	**	***	35
Arenaria bryophylla	Caryo	no	Ch	2	5			5	15	**	**	0	4			0	0			31
Alyssum klimesii	Brassica	no	H	0	0			2	1			1	1			7	15	*	*	27
Saussurea glacialis	Aster	no	H	0	1			0	0			6	17	*		1	2			27
Potentilla pamirica	Rosa	no	H	1	4			5	11			0	4	***	***	0	1			26
Dracocephalum heterophyllum	Lami	no	H	10	11			0	0			0	0			0	0			21
Carex sagaensis	Cyper	yes	H	2	3			0	2			4	8		**	0	0			19
Oxytropis chiliophylla	Faba	no	H	2	17	***	***	0	0			0	0			0	0			19
Saxifraga nanella	Saxifraga	yes	Ch	0	0			0	0			0	0			3	16	***	***	19
Stellaria depressa	Caryo	no	H	5	10		***	0	3			0	0			0	0			18
Saussurea hypsipeta	Aster	no	H	0	0			0	0			2	11	*	**	0	0			13
Draba oreades	Brassica	no	Ch	2	7			0	0			0	0			1	0			10
Elymus schrenkianus	Poa	yes	H	2	7		*	0	0			0	0			0	0			9
Aphragmus oxycarpus	Brassica	no	H	0	0			0	0			1	3			1	3			8
Astragalus confertus	Faba	no	H	0	0			0	8	***	***	0	0			0	0			8
Elymus schugnanicus	Poa	yes	H	3	4			0	0			0	0			0	0			7
Delphinium brunonianum	Ranuncul	no	H	0	5			0	0			0	0			0	0			5
Urtica hyperborea	Urtica	no	H	0	4			0	0			0	0			0	0			4
Festuca tibetica	Poa	yes	H	0	0			0	0			2	0			0	0			2
Saxifraga cernua	Saxifraga	yes	H	0	0			0	0			0	0			0	2			2
Carex sp.	Cyper	yes	H	0	0			1	0			0	0			0	0			1
Saussurea glanduligera	Aster	no	H	0	1			0	0			0	0			0	0			1
Stipa subsessiliflora	Poa	yes	H	0	1			0	0			0	0			0	0			1
	total			62	145	3	4	88	182	7	7	129	248	7	8	112	237	7	8	
	average richness			0.9	2.2			1.3	2.8			1.8	3.7			1.5	3.3			
	total richness			12	17			9	13			10	13			11	13			

**Figure 4:**
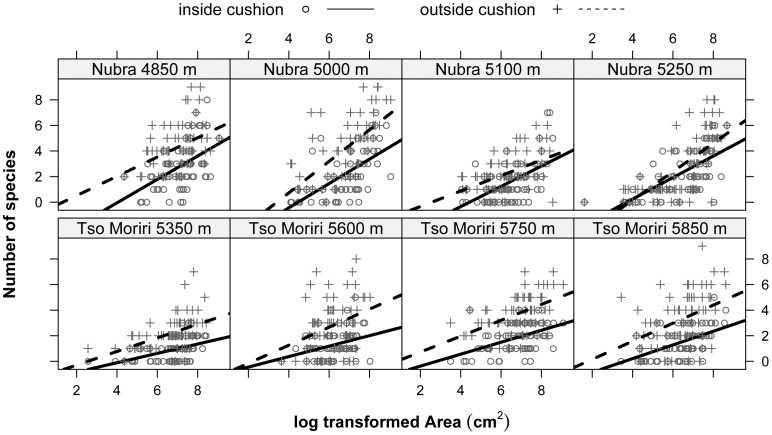
Species-area relationships for cushion and open areas on each study site. Statistics for each regression analysis are provided in Table S1.

The maximum number of species recorded within a single cushion or open area was 8 and 9, respectively. In all elevation sites, species richness increased with the sample area, both inside cushions and in the surrounding open areas (Figure 4). However, the intercepts of regression lines for cushions were always significantly smaller than those obtained for the open areas (Table S1), showing that an average open area at all sites always harboured more species than an average cushion. Across the entire elevation range of *T. caespitosum*, the numbers of species were significantly higher outside cushions (Figure 4) with the exception of Nubra 5250 m, where there were nonsignificant diferences in species richness (Table S1). Slopes of regression lines showed no difference between the two habitats in all study sites, indicating that the difference in species richness between the cushions and open areas were constant across the sample sizes.

**Figure 5:**
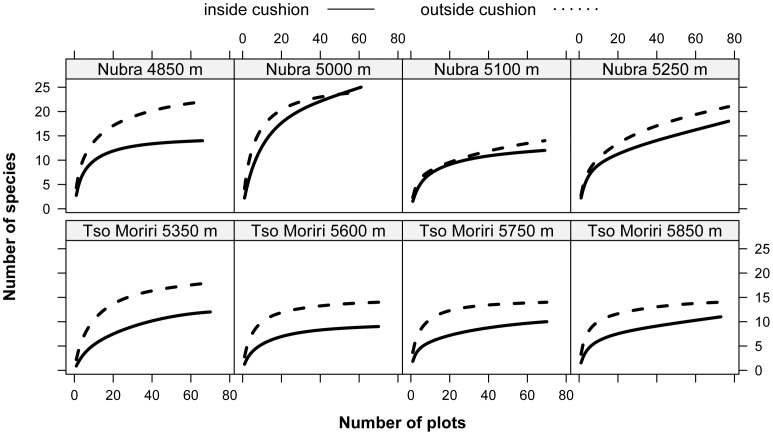
Sample-based rarefaction curves for cushions and open areas on each study site. Note an increase in the number of species encountered with increasing number of plots sampled. Open areas outside cushions are more species rich than equal areas inside cushions at all elevations.

The total species richness at the maximal number of samples, estimated from the sample-based rarefaction curves (Figure 5), followed the same pattern as the local richness, with fewer species inside the cushion. However, the magnitude of these differences varied among the study sites. In Nubra, the differences between the two habitats were small (on average 8.1% more species in the open areas than cushions), with the exception of the lowest (driest) site in 4850 m (36% more species in the open areas). In Tso Moriri, open areas in all four elevation sites contained significantly more species than cushions (on average 30% more species in the open areas).

### Vegetation Composition and Habitat Preferences of Individual Species

**Figure 6:**
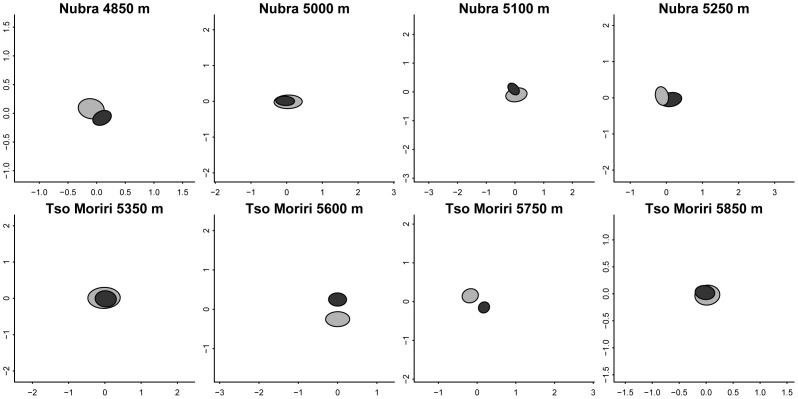
Differences in species composition of vascular plants between the cushions (black) and open areas outside cushions. Diagrams are from non-metric multidimesional scaling (NMDS). Each diagram shows 95% confidence ellipses around multivariate centroid of samples from cushions (black) and open areas outside cushions (grey). Differences are statistically significant (p<0.01) at all elevations (detailed results from PERMANOVA are given in Table S2).

In all study sites, the species composition of vascular plants growing on cushions differed significantly from that in open areas (Table S2), although the magnitude of compositional dissimilarity also varied between the sites. This was indicated by different degrees of overlap between the confidence intervals of centroids for cushions and open areas in the NMDS ordination diagrams (Figure 6). In Nubra, 15 species (39.5%) showed a preference for a habitat and all of these species preferred open areas (Table 2). In the case of *Poa attenuata*, the preference was consistent over all four elevational sites. *Astragalus strictus* and *A. confertus* preferred open areas in all of the three sites they occurred in. *Potentilla pamirica* preferred open areas at the three lower sites but not at the highest site. In Tso Moriri, 18 species (60%) showed a preference and all of these species again preferred open areas (Table 3). *Poa attenuata, Draba altaica, Eritrichium hemisphaericum* and *Saussurea gnaphalodes* preferred open areas at three sites. The least number of species having a significant habitat preference for areas outside cushions was found in Nubra 5250 m, while the highest numbers of species prefering open areas occurred in the two subnival sites in Tso Moriri 5700–5850 m, and in the steppe site in Nubra 5000 m. There were species with the same occurrence pattern but different statistical significance of habitat preference; this was because of differences in cover-abundance data between the cushion and the outside plot.

### Outcome of Plant–plant Interactions

**Figure 7:**
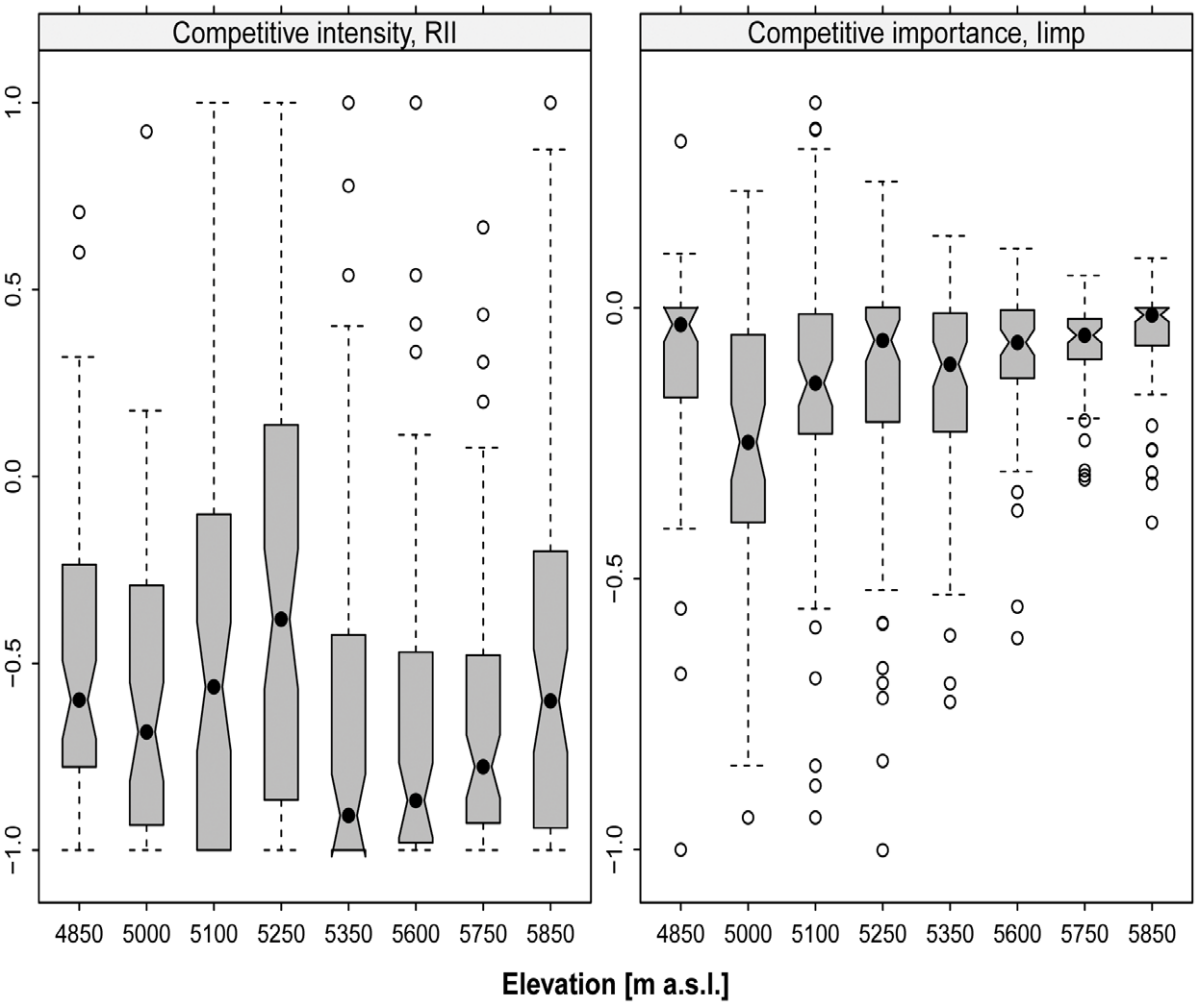
Outcome of plant–plant interactions at community level on each study site in Nubra (4850–5250 m) and Tso Moriri (5350–5850 m). Shown are the competitive intensity and competitive importance values between *T. caespitosum* and other species. Responses were calculated such that competition is represented by negative values and facilitation by positive values. Individual species responses to the presence of cushions are given in Figure S1.

The RII and I_imp_ indices calculated at community level revealed that the effects of *T. caespitosum* cushions on neighbouring plants were significantly negative at all elevations at both Nubra and Tso Moriri (Figure 7). This suggests the prevalence of competitive interactions. RII values were significantly lower in Tso Moriri when compared with Nubra, indicating more intense competition in more extreme elevations. In both localities, however, there was a tendency for competitive intensity and importance to diminish with increasing elevation (less negative values towards higher elevations, Figure 7). Species-specific responses to the presence of cushions were negative in 78% of tested relationships (19 positive responses out of 86 analyzed in total) in Nubra, and 92% in Tso Moriri (5 positive interactions out of 63 in total) (Figure S1). Most species with positive RII and I_imp_ values had a low (<5 records) frequency of occurrence (Table 2 and 3), and none of them showed a significant habitat preference in the Friedman-based permutation test.

### Microclimate

In Nubra the vegetation season, defined as the period with mean daily temperatures above freezing, lasted between three months (mid May to beginning of September ) at 5250 m and five months (the beginning of May to mid October) at 4850 m. The mean air annual/summer temperatures decreased from −1.6/7.7°C to −3.6/7.1 and the relative air humidity increased from 39/38% to 87/69% with increasing elevation. At all sites, air temperatures in the warmest month of August remained above zero both day and night. Compared with the similar elevation in Tso Moriri, a wetter Nubra site at 5250 m had ∼two months shorter growing season due to deeper snowpack which persisted longer into the spring (personal observation). This leads to a higher water supply and the development of alpine grasslands compared to the dry steppes in Tso Moriri. This was also evident from differences in winter temperatures, which were higher and less fluctuating in Nubra than in Tso Moriri due to more effective snow protection (mean ± S.D.: −7.9±0.7°C vs −16.8±3.1°C during January-February).

In Tso Moriri, the vegetation season lasted nearly five months at 5350 m, 3.5 months at 5600 m, and three months at 5750 m. At the highest elevation, the vegetation season was restricted to less then two months (Table 3). Even during August, when temperatures generally reached their highest values, temperatures regularly dropped below freezing during the night; at the highest sites repeatedly to about −5°C. The sites differed mainly in the duration of the sub-zero temperature spells over the course of a 24-hour period. While air temperature 10 cm above ground never dropped below zero at the lowest elevation during August, it usually fell below zero for about 2–3 hours at the middle elevations; at the highest elevation at 5850 m freezing lasted between 5 and 10 hours every day, particularly in the second half of August when many plants still flowered and fruited. Daily air temperatures rose to 15–20°C at all four elevations but for a much shorter time per day at higher elevations. The mean air annual/summer temperatures decreased from −4.4/7.3°C and −10.4/4.4°C between 5350–5850 m, while relative air humidity increased from 61/50% to 84/53%.

**Figure 8:**
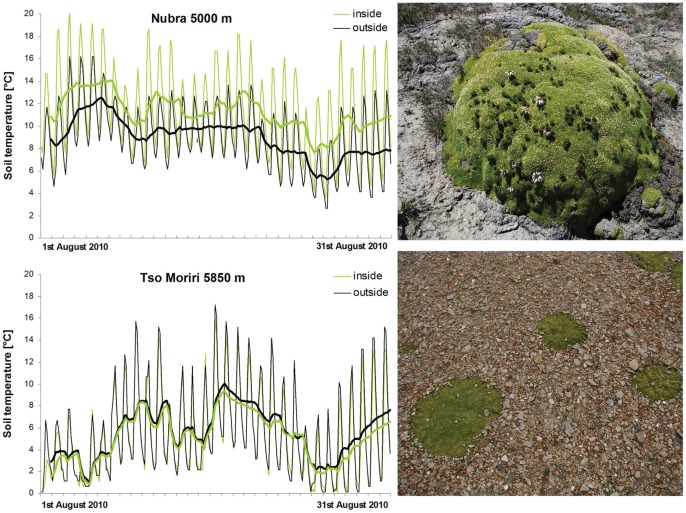
Soil temperature regime. Sampled inside the *T. caespitosum* cushion and in the soil of the adjacent open area at Nubra 5000 m and Tso Moriri 5850 m. Thick lines depict the moving average for eight daily measurements. Photos on the right side of the panel show the variability in the growth of cushions; generally, individuals from lower elevations are gibbous and protruding above the ground, individuals from the upper part of the distributional gradient are flat.

Cushions from a low site in Nubra (5000 m) provided warmer microsites, as measured 2-cm below ground, compared with open areas (annual mean T = −0.3°C vs. −3.4°C). They also had twice as many degree-days (1227 vs. 601 at T_base_ = 0°C) and a frost-free period lasting a month longer (Table 3). On the other hand, the differences at the highest site in Tso Moriri were only minor, the open areas being even slightly warmer than cushions (Table 4, Figure 8).

**Table 4:** Soil temperature regime and degree days (°d) at 2–3 cm depth inside and outside cushions.

		Nubra 5000 m		Tso Moriri 5600 m		Tso Moriri 5850 m	
		in	out	in	out	in	out
Whole year	Tmean	−0.3	−3.4	−6.7	−6.4	−7.4	−7.4
	Tmax	20.2	16.2	19.2	15.2	16.2	17.2
	Tmin	−15.6	−15.5	−22.1	−20.5	−25.6	−26.1
	daily Tmean>0°C	8.4.–14.10.	5.5.–10.10.	5.6.–27.9.^a^	2.6.–5.10.^b^	20.6.–27.9.^c^	20.6.–24.9.^d^
	frost-free period	9.4.–10.10.	5.5.–9.10.	4.7.–25.8.	20.6.–4.9.	21.6.–3.9.	3.7.–2.9.
	°d (Tbase = 0°C)	1227	601	434	391	330	351
	°d (Tbase = 3°C)	736	329	196	162	123	137
Growing season	Tmean	7.7	4.0	3.1	2.7	2.1	2.3
(15.V.–15.IX.)	Tmax	20.2	16.2	19.2	15.2	16.2	17.2
	Tmin	0.6	0.1	−10.0	−7.9	−8.9	−11.0
	°d (Tbase>0°C)	949	500	427	385	326	349
	°d (Tbase>3°C)	600	294	196	162	123	137

Measured in 3-hour intervals from 1.9.2009 to 31.8.2010. Values with the lowercase superscript letter indicate situation when there was even an earlier day with Tmean>0 on the beginning of the vegetation season (a - 2.6., b - 28.5., c - 6.5., d - 8.6.); these early days however were often followed by a significant period of days with Tmean<0, that is why a later date is used in the table so that it better represents the beginning of the vegetation season. During the frost-free period no freezing temperatures were recorded, even if mild soil freezing might have occurred.

### Soil

The contents of NH_4_^+^-N, PO_4_^3–^P values were higher in the soil of open areas at both localities (Table 5). PO_4_-P and K content significantly decreased with elevation while the Na content increased with elevation at both localities. In Nubra only, more Mg was found in the soil of open areas, while the OM content was higher in the soil below cushions, significant at 5000 and 5100 m elevations. The OM and concentration of NH_4_^+^-N decreased with elevation. Some interactions were revealed: the NO_3_^–^N content increased inside the cushions and decreased in the open areas with increasing elevation. Soil water content was low at both localities, from 2 to 17%; the VWC in Nubra was higher outside the cushions, significantly at 5100 and 5250 m elevations, and increased in the open areas and decreased inside the cushions with increasing elevation.

**Table 5:** Soil physico-chemical characteristics.

		Nubra						Tso Moriri					
		4850	5000	5100	5250	Cushion	Elevation	5350	5600	5750	5850	Cushion	Elevation
N-NH^4+^ mg/kg	In	1.7	2.6	1.7	1.4	*out>in	*↓	1.1	1.0	1.1	1.1	***out>in	
	Out	**2.4**	2.3	2.0	**1.8**			**2.0**	**1.8**	**1.8**	**2.1**		
N-NO^3-^ mg/kg	In	0.5	0.8	**2.7**	**1.3**		**↑in↓out	1.2	0.7	0.9	0.6	*out>in	**↓
	Out	**1.4**	**1.2**	1.0	0.9			**2.7**	1.0	1.2	**1.2**		
TN mg/kg	In	487	1686	1163	985			833	697	1252	1415		**↑
	Out	613	1385	778	1024			738	957	1148	1554		
P-PO_4_^3-^mg/kg	In	17.6	19.1	12.6	12.3	*out>in	***↓	28.2	14.1	24.8	12.7	*out>in	*↓
	Out	**23.0**	**25.7**	14.4	13.0			**39.5**	**20.8**	23.1	17.2		
Ca mg/g	In	5.2	32.6	12.7	17.7			2.9	2.5	2.4	2.5	*out>in	**↓in↑out
	Out	7.3	33.3	17.3	17.6			2.5	3.0	**3.0**	**3.6**		
Mg mg/g	In	8.1	8.5	7.6	7.5	*out>in		3.5	2.8	2.5	2.2		***↓
	Out	7.6	**9.9**	**8.6**	8.3			3.1	3.1	2.6	2.5		
K mg/g	In	6.5	3.7	3.9	3.7		***↓	3.0	1.8	2.2	1.9		*↓greater in
	Out	5.9	4.4	4.0	4.1		***↑	2.4	2.0	2.2	2.2		
Na mg/g	In	0.6	0.6	0.9	0.9			0.3	0.2	0.5	0.5		***↑greater out
	Out	0.4	0.7	0.9	0.9			0.1	0.2	0.6	0.6		
OM %	In	2.4	**7.2**	**4.4**	2.8	**in>out		2.3	2.0	2.6	2.8		
	Out	1.8	3.0	2.7	2.9			2.5	2.2	2.3	2.8		
GWC %	In	3.9	1.1	1.2	1.7	***out>in	**↓	2.8	2.8	2.1	2.7		
	Out	**7.0**	2.2	2.2	2.4			3.7	2.8	3.0	2.6		
VWC %	In	10.8	11.2	10.5	7.8	***out>in	*↓in↑out	**7.2**	**8.5**	9.1	**12.2**	***in>out	***↑greater in
	Out	13.2	13.3	**15.0**	**14.0**			4.7	4.7	5.8	5.0		
Soil particles>0.5 mm %	In	54.0	19.9	14.4	42.5		*↓	11.9	10.6	15.1	20.1		**↑
	Out	55.4	25.9	18.0	35.9			13.3	10.2	13.4	18.6		
pH	In	8.4	8.5	8.6	8.4	**out>in		7.7	7.7	7.1	6.9	***out>in	**↓
	Out	8.5	8.8	**9.0**	**8.8**			**8.1**	**8.3**	**8.0**	**7.8**		

Availability of soil nutrients in the presence (in) or absence (out) of *T. caespitosum* cushions. An upward or downward pointing arrow indicates a positive or negative relationship between the dependent variable and elevation, based on the likelihood-ratio test (*** P<0.001, ** P<0.01, * P<0.05). Also shown are post hoc Tukey tests on paired differences (significantly higher values inside/outside cushion are in bold).

In Tso Moriri only, NO_3_^–^N and Ca contents were higher in the soil of open areas, most significantly at 5350 and 5850 m elevations. Considering the trends with elevation irrespective of the habitat, the contents of NO_3_^–^N and Mg in the soil decreased, while the total N increased (Table 5). Some interactions were also revealed: the Ca content in the soil decreased with elevation inside the cushions and increased in the open areas. The pH and K content in the soil decreased with elevation. This decrease was greater inside the cushions. The VWC in Tso Moriri was higher inside the cushions and this difference increased with elevation. The Na content increased with elevation and this increase was greater in the open areas.

## Discussion

It is generally assumed that the diversity and composition of plant communities is determined by a species pool (i.e. a set of specieś propagules which are able to reach the site), an environmental filter (environmental restrictions on species survival) and a community filter (biotic interactions, mainly competition) [53]. The species pool size is correlated with the surrounding area of a given habitat [54] and hence its effect on local species richness is relatively straightforward. The effect of the community filter is probably the least predictable one – each species is unique. The presence of a strong competitor, which is able to dominate the community and have an impact on other species, is difficult to predict and may have historical and/or environmental reasons. SGH, in particular, predicts that strong competitors will occur with higher probability in favourable environments having ample resources. These are less likely in stressful environments, e.g. at higher elevations, where neutral interactions or even facilitation by dominant plants may prevail over competition [19].

We investigated the association pattern of plants with the dominant cushion plant *T. caespitosum* and assessed its influence on microclimate and soil physico-chemical properties in communities along an elevational gradient in a dry region of the Indian Trans-Himalaya. Our hypotheses were based on the fact that cushions are not only well adapted to the harsh climate of high mountains but, owing to their specific characteristics, are also able to positively modify microsite conditions. This helps other species establish, survive or perform better. There are about two dozen studies focusing on this topic, with practically all of them supporting this idea [8], [9], [12]–[17]. With just a few exceptions [22], cushion plants were proven to facilitate other plant species and enhance the local plant richness and diversity. The facilitation was more prominent in more stressful environments, such as relatively high elevations, indicating the validity of SGH. Our study, however, shows something rather different. We attribute this to a combination of extreme elevation and aridity in our study area: cushions do not provide better microsites for other plants under these conditions.

### Microclimate and Soil Nutrients

In lower elevations, cushions increased soil temperature and prolonged the growing season in comparison with the adjacent open areas. These differences, however, diminished with elevation such that the open substrates even tended to be warmer at the highest elevations; this was also observed by Cavieres et al. [55]. Badano et al. [26] noticed that cushions of *Azorella monantha* maintained a lower temperature than the open area for much of the time, as was the case at our uppermost site. At Nubra (5000 m) and Tso Moriri (5600 m), there was a wider variation in temperature within cushions than in the open area while we expected the opposite to be true [8].

The nursing effect of cushions, besides the other mechanisms mentioned above, is often attributed to better nutrient availability in the soil below them [8]–[10]. Our results contradict these findings. Soil the PO_4_^−3^-P and NH_4_^+^-N contents were lower in the soil below cushions at both localities. Moreover, K decreased with elevation and the decrease was greater below the cushions. Additionally, the soils below cushions contained less Mg in Nubra and less NO_3_^–^N and Ca in Tso Moriri. Only the higher content of OM in soils below cushions (in Nubra only) was consistent with earlier reports [9]. The markedly high contents of Mg and K in soils from Nubra are caused by the specific geochemistry of the Karakorum batholith (rich in Mg) and leucogranites (rich in K) [35]. The main source of enhanced nutrient levels in the soil below cushions is the decomposing dead tissue accumulated within them [10]. Such a decomposition, however, is presumably rather slow in the very dry and cold climate of the Trans-Himalaya and may not be sufficient to replenish the nutrients utilised by the cushion itself. Cushions, therefore, do not always provide a microsite with better nutrient availability. Moreover, the difference in nutrient concentration might also be caused by the activity of biological soil crusts [56], which commonly occurred in the open areas of our study region. These are known for their capability of raising nutrient levels [57], [58].

### Plant-plant Interactions

Since plants compete for the same resources, there would have to be a strong reason for other species to prefer growing inside cushions. We could not prove that cushions ameliorated microsite conditions, a fact which would overshadow the competive pressure. Our results, on the contrary, showed that there were less nutrients in the soil under cushions and a temperature regime comparable to open areas. The only exception was a lower site in Nubra, where the cushion increased the soil temperature below it, however, the plants on the top of the cushions might still have suffered from overheating. Furthermore, *T. caespitosum* cushions are exceptionally hard and compact and this will naturally reduce the opportunity for other plants to establish themselves inside. This brings us to realisation that the observed negative plant-plant interactions might be a result of "cushion quality effect" rather than a climate effect, and that comparison with other cushion species growing in the same environment is needed [27]. In fact, the probability of being colonised differs among cushion species, which are usually included in various comparisons and generalisations. Typical cushions, e.g. *Azorella* spp., *Laretia acaulis* or *Arenaria polytrichoides*, are often compared to species with relatively loose branches, which allows interacting species to establish more easily. This indicates also that the term “cushion plant“ is ambiguously understood, at least in this point of view, but this issue is discussed only rarely [27].

Our results showed that along the entire elevation range of *T. caespitosum*, the open areas were not only richer in species composition, but not a single species preferred to grow inside cushions. This preference for the habitat outside cushions is in sharp contrast to the results from other studies on cushions [59]–[68]. In addition to the fact that the cushions did not provide a better microhabitat, we ascribe the prevailing negative association between cushions and other plants to the extreme conditions present in our study area.

In the Tso Moriri region, vascular plants grow at one of the highest elevations in the world, occurring up to 6150 m. This is due to the relatively flat terrain of an unglaciated high-elevation plateau bearing well-developed soils. The current record (6400 m, *Saussurea gnaphalodes*) [69] is actually from a much wetter region of Mt. Everest, where the circumstances of the find indicate that the plant was rather isolated in an exceptionally suitable microhabitat. This is not the case in our study region, where the vegetation is continuous up to 5960–6000 m, consisting of 9 species here, although with a negligible cover [33]. *T. caespitosum* and accompanying species reach their absolute elevational limit here. On the very margins of angiosperm existence, the conditions are so extreme that species inhabiting such places must be stress-tolerant and well adapted in order to survive on their own. Moreover, the cushions at high elevations are mostly flat and do not protrude much above the ground, which reduces their possible sheltering effect against desiccating wind and abrasion. Thus, other plants might preferentially establish and grow in protected microsites in the lee of the countless stones instead of the cushions.

The observed trend of species preferring open areas was also consistent in the lower parts of the gradient both in Nubra and Tso Moriri. Although the cold steppes zone here has a more favourable temperature regime, from air relative humidity measurements and the apparent precipitation increase with elevation in combination with uneven evapotranspiration rates, we assume that plants from lower elevations are subject to higher water stress. This assumption was also supported by our pilot study on plant transpiration rates (Doležal et al. unpublished). All the species growing here must be well adapted to the arid conditions with water as a limiting factor. Therefore, the species did not need a cushion to survive and preferred the open areas where one can expect lower competition for water.

The negative plant-plant interactions prevailing across the entire elevation range of *T. caespitosum* from 4850 to 5850 m can be explained by the competition for water and nutrients together with negligible or no advantage provided by cushions. The less negative association observed at the upper part of Nubra Valley at 5250 m seems to be linked to less stresfull water and thermal conditions, as indicated by the prevalence of alpine grasslands with high cover. We suppose, however, that this pattern is a result of cushions being more prone to invasion by other species (those belonging to alpine grassland such as *Poa attenuata, Carex pseudofoetida, Astragalus strictus*; Table 2) rather than a result of facilitation. *Thylacospermum caespitosum* is a strong competitor in highly stressful conditions, while in mesic conditions (with higher frequency of fast-growing clonal and hence competitive species like grasses and sedges) it is more prone to invasion. The colonisation by other species can be further enhanced by small cracks in cushions caused by abundant yaks which graze on alpine grasslands during the whole summer season.

The observed patterns of plant interactions in this study can be put into the context of ongoing postglacial successional changes in the Himalayas. Our studied cushion species can be called a long-lived pioneer; deep roots allow it to colonise infertile glacial substrata early after deposition and then occupy the spot for long time periods due to its slow vegetative spread (unpublished data). The cushions can dominate succession for a very long time, in particular on a stressful site where primary succession towards closed-canopy vegetation, like alpine grassland, is blocked either because the site is dry (low-elevation cold steppes) or very cold (high-elevation subnival zone). If the site is not extremely stressful, *T. caespitosum* is replaced by other species as seen around the old glacial lakes in the upper part of the Nubra locality, where the final stages of postglacial succession are alpine grasslands dominated by graminoids.

## Supporting Information

Figure S1 Intensity of interactions between *T. caespitosum* and other species. Calculated using the relative interaction index, competition is represented by negative values and facilitation by positive values. Error bars represent standard errors.
(TIF)

Table S1 Test of species-area relationships. Explained variability (adjusted R^2^) from the regressions of log (number of species) (inside and outside cushion) on log (sample area) (i.e. cushion size) in eight elevational sites in Nubra (4850–5250 m) and Tso Moriri (5350–5850 m), with corresponding Type I error estimate (n.s. nonsignificant, ^a^ *P*<0.1, *P<0.05, **P<0.01, ***P<0.001). Shown are also tests of differences in slope and intercept parameter estimates between the two regression lines.
(DOC)

Table S2 PERMANOVA results. Permutational multivariate analysis of variance testing for the differences in species composition between the cushion habitat and open areas.
(DOC)
