# Supplementary figures and images for: Testing the Stress-Gradient Hypothesis at the Roof of the World: Effects of the Cushion Plant Thylacospermum caespitosum on Species Assemblages
Source: PLoS One. 2013 Jan 10;8(1):e53514. doi: 10.1371/journal.pone.0053514 (PMC3542354; doi:10.1371/journal.pone.0053514)

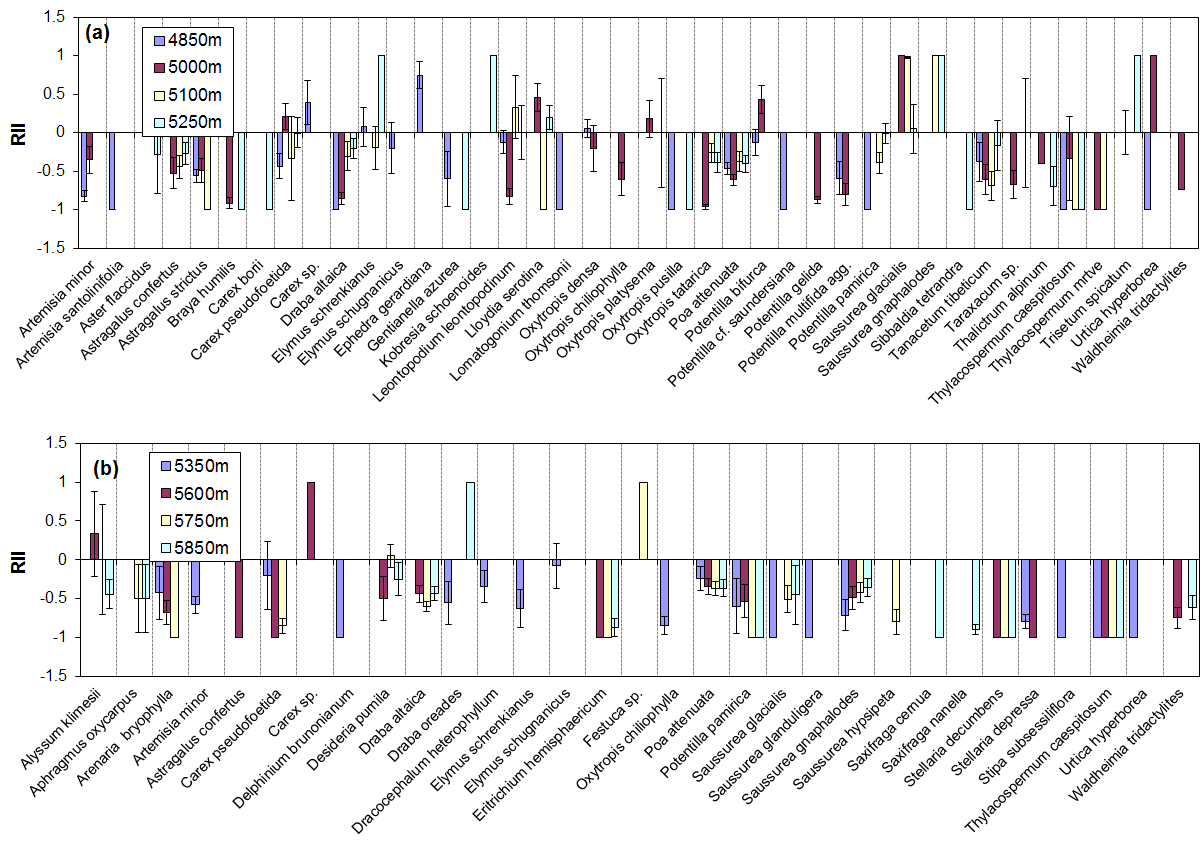

Supplement: Figure S1 — Intensity of interactions between T. caespitosum and other species. Calculated using the relative interaction index, competition is represented by negative values and facilitation by positive values. Error bars represent standard errors. (TIF) [file pone.0053514.s001.tif]
