# Supplementary material for: Testing the Stress-Gradient Hypothesis at the Roof of the World: Effects of the Cushion Plant Thylacospermum caespitosum on Species Assemblages
Source: PLoS One. 2013 Jan 10;8(1):e53514. doi: 10.1371/journal.pone.0053514 (PMC3542354; doi:10.1371/journal.pone.0053514)
Supplement: Table S1 — Test of species-area relationships. Explained variability (adjusted R2) from the regressions of log (number of species) (inside and outside cushion) on log (sample area) (i.e. cushion size) in eight elevational sites in Nubra (4850–5250 m) and Tso Moriri (5350–5850 m), with corresponding Type I error estimate (n.s. nonsignificant, a P<0.1, *P<0.05, **P<0.01, ***P<0.001). Shown are also tests of differences in slope and intercept parameter estimates between the two regression lines. (DOC) [file pone.0053514.s002.doc]

**Supporting Information**

**Table S1.**

| *Nubra* | *Inside* | *Outside* | *different slopes* | *different intercepts* |
| --- | --- | --- | --- | --- |
| 4850 | 15.01** | 18.2*** | a | *** |
| 5000 | 23.48*** | 40.79*** | n.s. | *** |
| 5100 | 25.61*** | 29.7*** | n.s. | ** |
| 5250 | 31.97*** | 59.38*** | n.s. | n.s. |
| *Tso Moriri* |  |  |  |  |
| 5350 | 20.16** | 28.68*** | n.s. | *** |
| 5600 | 8.51* | 5.92* | n.s. | *** |
| 5750 | 3.77* | 17.67*** | n.s. | *** |
| 5850 | 7.77* | 12.27** | n.s. | *** |

**Table S2.** Results of permutational multivariate analysis of variance (PERMANOVA) testing for the differences in species composition between the cushion habitat and open areas.

|  |  | *D.f.* | *Sums Of Sqs* | *Mean Sqs* | *F.Model* | *R^2^* | *Pr(>F)* |  |
| --- | --- | --- | --- | --- | --- | --- | --- | --- |
| Nubra 4850 m | Cushion | 1 | 2.1 | 2.1 | 7.8 | 0.062 | 0.001 | *** |
|  | Residuals | 118 | 31.2 | 0.3 | 0.9 |  |  |  |
|  | Total | 119 | 33.2 | 1.0 |  |  |  |  |
| Nubra 5000 m | Cushion | 1 | 1.3 | 1.3 | 3.6 | 0.037 | 0.001 | *** |
|  | Residuals | 92 | 32.4 | 0.4 | 1.0 |  |  |  |
|  | Total | 93 | 33.6 | 1.0 |  |  |  |  |
| Nubra 5100 m | Cushion | 1 | 0.6 | 0.6 | 2.0 | 0.023 | 0.012 | * |
|  | Residuals | 86 | 26.0 | 0.3 | 1.0 |  |  |  |
|  | Total | 87 | 26.7 | 1.0 |  |  |  |  |
| Nubra 5250 m | Cushion | 1 | 0.8 | 0.8 | 2.4 | 0.024 | 0.001 | *** |
|  | Residuals | 100 | 30.7 | 0.3 | 1.0 |  |  |  |
|  | Total | 101 | 31.5 | 1.0 |  |  |  |  |
| Tso Moriri 5350 m | Cushion | 1 | 0.9 | 0.9 | 2.3 | 0.028 | 0.002 | ** |
|  | Residuals | 82 | 30.4 | 0.4 | 1.0 |  |  |  |
|  | Total | 83 | 31.3 | 1.0 |  |  |  |  |
| Tso Moriri 5600 m | Cushion | 1 | 1.1 | 1.1 | 3.9 | 0.045 | 0.001 | *** |
|  | Residuals | 82 | 23.9 | 0.3 | 1.0 |  |  |  |
|  | Total | 83 | 25.1 | 1.0 |  |  |  |  |
| Tso Moriri 5750 m | Cushion | 1 | 2.4 | 2.4 | 10.2 | 0.082 | 0.001 | *** |
|  | Residuals | 114 | 27.2 | 0.2 | 0.9 |  |  |  |
|  | Total | 115 | 29.7 | 1.0 |  |  |  |  |
| Tso Moriri 5850 m | Cushion | 1 | 1.2 | 1.2 | 4.1 | 0.038 | 0.001 | *** |
|  | Residuals | 102 | 29.2 | 0.3 | 1.0 |  |  |  |
|  | Total | 103 | 30.3 | 1.0 |  |  |  |  |

**Figure S1.** Intensity of interactions between *Thylacospermum* and other species in different elevations of Nubra (4850-5250 m) and Tso Moriri (5350-5850 m) as calculated with the relative interaction index (RII; Armas et al. 2004). Responses were calculated such that competition is represented by negative values and facilitation by positive values. Error bars represent standard errors.
