# Supplementary material for: Testing the Stress-Gradient Hypothesis at the Roof of the World: Effects of the Cushion Plant Thylacospermum caespitosum on Species Assemblages
Source: PLoS One. 2013 Jan 10;8(1):e53514. doi: 10.1371/journal.pone.0053514 (PMC3542354; doi:10.1371/journal.pone.0053514)
Supplement: Table S2 — PERMANOVA results. Permutational multivariate analysis of variance testing for the differences in species composition between the cushion habitat and open areas. (DOC) [file pone.0053514.s003.doc]

**Table S2.**

|  |  | *D.f.* | *Sums Of Sqs* | *Mean Sqs* | *F.Model* | *R^2^* | *Pr(>F)* |  |
| --- | --- | --- | --- | --- | --- | --- | --- | --- |
| Nubra 4850 m | Cushion | 1 | 2.1 | 2.1 | 7.8 | 0.062 | 0.001 | *** |
|  | Residuals | 118 | 31.2 | 0.3 | 0.9 |  |  |  |
|  | Total | 119 | 33.2 | 1.0 |  |  |  |  |
| Nubra 5000 m | Cushion | 1 | 1.3 | 1.3 | 3.6 | 0.037 | 0.001 | *** |
|  | Residuals | 92 | 32.4 | 0.4 | 1.0 |  |  |  |
|  | Total | 93 | 33.6 | 1.0 |  |  |  |  |
| Nubra 5100 m | Cushion | 1 | 0.6 | 0.6 | 2.0 | 0.023 | 0.012 | * |
|  | Residuals | 86 | 26.0 | 0.3 | 1.0 |  |  |  |
|  | Total | 87 | 26.7 | 1.0 |  |  |  |  |
| Nubra 5250 m | Cushion | 1 | 0.8 | 0.8 | 2.4 | 0.024 | 0.001 | *** |
|  | Residuals | 100 | 30.7 | 0.3 | 1.0 |  |  |  |
|  | Total | 101 | 31.5 | 1.0 |  |  |  |  |
| Tso Moriri 5350 m | Cushion | 1 | 0.9 | 0.9 | 2.3 | 0.028 | 0.002 | ** |
|  | Residuals | 82 | 30.4 | 0.4 | 1.0 |  |  |  |
|  | Total | 83 | 31.3 | 1.0 |  |  |  |  |
| Tso Moriri 5600 m | Cushion | 1 | 1.1 | 1.1 | 3.9 | 0.045 | 0.001 | *** |
|  | Residuals | 82 | 23.9 | 0.3 | 1.0 |  |  |  |
|  | Total | 83 | 25.1 | 1.0 |  |  |  |  |
| Tso Moriri 5750 m | Cushion | 1 | 2.4 | 2.4 | 10.2 | 0.082 | 0.001 | *** |
|  | Residuals | 114 | 27.2 | 0.2 | 0.9 |  |  |  |
|  | Total | 115 | 29.7 | 1.0 |  |  |  |  |
| Tso Moriri 5850 m | Cushion | 1 | 1.2 | 1.2 | 4.1 | 0.038 | 0.001 | *** |
|  | Residuals | 102 | 29.2 | 0.3 | 1.0 |  |  |  |
|  | Total | 103 | 30.3 | 1.0 |  |  |  |  |
